# Reported child awareness of parental depression

**DOI:** 10.1192/pb.bp.113.044198

**Published:** 2014-06

**Authors:** Olga Eyre, Rhys Bevan Jones, Becky Mars, Gemma Hammerton, Ruth Sellers, Robert Potter, Ajay Thapar, Frances Rice, Stephan Collishaw, Anita Thapar

**Affiliations:** 1 Child and Adolescent Psychiatry Section, Institute of Psychological Medicine and Clinical Neurosciences, School of Medicine, Cardiff University, UK; 2 School of Social and Community Medicine, University of Bristol, UK; 3 Cwm Taf Health Board, UK; 4 Taff Riverside Practice, Cardiff, UK; 5 Department of Clinical, Educational and Health Psychology, University College London, UK

## Abstract

**Aims and method** To determine rates of parent-reported child awareness of parental depression, examine characteristics of parents, children and families according to child awareness, and explore whether child awareness is associated with child psychopathology. Data were available from 271 families participating in the Early Prediction of Adolescent Depression (EPAD) study, a longitudinal study of offspring of parents with recurrent depression.

**Results** Seventy-three per cent of participating children were perceived as being aware of their parent’s depression. Older children, and children of parents who experienced more severe depression, were more likely to be aware. Awareness was not associated with child psychopathology.

**Clinical implications** Considering children in the context of parental depression is important. Child awareness may influence their access to early intervention and prevention programmes. Further research is needed to understand the impact of awareness on the child.

Are children aware of their parent’s depression? Which children are aware? Does awareness have an impact on them? Understanding communication with children about parental mental illness is of relevance to many families. Mental illness in parents is common, with about one in five young people being exposed to a parent with a mental illness.^1^ Depression is one of the most common mental disorders, being particularly prevalent during the child-bearing years.^2^ Prevalence estimates range from 6 to 17%^3^ and about 1 in 5 will experience major depression as parents.^4^ Parental depression increases risk for behavioural, developmental and emotional difficulties in the child^5^ and rates of disorder have been found to be three times higher in children of parents with depression.^6^

Rates of child awareness of parental depression are uncertain, as are characteristics of children and families who are aware. Some information about child awareness has been gained through qualitative studies exploring children’s experience of parent mental illness. Evidence from these studies is mixed regarding the level of knowledge and understanding children have, and how much information they need with regard to their parent’s mental illness.^7^ Children, parents and professionals often having differing views.^8^ However, overall, findings suggest children’s knowledge of their parent’s illness is often inadequate or inaccurate.^7^

For a parent, talking to a child about their depression may be difficult. Although it has been suggested that parents want their children to understand more about their mental illness,^7^ they may be reluctant to talk about it due to fear of stigma, feelings of guilt and shame^9^ or out of a desire to protect them from unnecessary burden or feelings of responsibility.^10^ Although few studies have examined the impact of child awareness of parental depression, the existing evidence does not support these parental concerns. Beardslee *et al*^11^ used a family-based intervention encouraging parents and children to talk about the effects of parental depression. They found children’s internalising scores decreased progressively following the intervention. Findings do suggest that children who are knowledgeable regarding their parent’s mental illness may be better able to cope.^7^ In addition, awareness of parental depression may allow early intervention and prevention strategies to be accessed by these children who are at risk of depression themselves.

This study aims to utilise an existing sample of parents with recurrent depression and their offspring to further explore the area of child awareness of parental depression. The specific aims were to:

- establish rates of parent-reported child awareness of parental depression
- examine characteristics of families according to child awareness
- explore whether child awareness is associated with child psychopathology.

## Method

### Participants

The sample consisted of families taking part in the Early Prediction of Adolescent Depression (EPAD) study. This is a three-wave longitudinal study of 337 parents with a history of recurrent unipolar depression (at least two episodes confirmed at interview), together with their offspring. Parents were recruited predominantly from primary care practices in South Wales, UK. Parents with bipolar disorder or psychotic disorder were excluded. Children were aged 9-17 years at entry, biologically related to the affected parent, and had IQ test scores of ⩾50. The study is described in detail elsewhere.^12^ At the final follow-up, parents completed a questionnaire asking them who knew about their depression. Of the 337 parents initially recruited, 309 took part in the final follow-up, of whom 271 completed the questionnaire on awareness of their depression (88%). There were no differences between those who completed the questionnaire and those who did not on most variables examined (parent age and gender, child age and gender, family composition and income, parent depression severity and rates of disorder in the child). The exceptions were child IQ (*t*(303) = 1.93, *P* = 0.055) and parent education level (χ^2^ = 4.45, d.f. = 1, *P* = 0.035), which were lower in those who did not complete the questionnaire. The parent sample consisted of 18 fathers and 253 mothers (28-58 years, mean 44). The child sample consisted of 114 males and 157 females (10-19 years, mean 14.8).

### Procedure

Families were visited at home at three time points between April 2007 and April 2011. At each wave of data collection, informed consent was obtained and both parent and child were interviewed independently by two trained psychology graduates. Parent and child questionnaires were completed.

### Measures

#### Parent measures

During the final assessment (wave three), the index parent was asked to indicate from a list of individuals (including spouse/partner, study child, another child, parent, sibling, friend, colleague, other) who they perceived was aware of their depression. Parent demographic information including education level (dichotomised as university or above) and income (dichotomised according to the sample median of £30 000) were collected via questionnaire at each time point. Data from the final follow-up (wave three) were used in the current study. Information regarding history of parental depression was obtained at baseline by compiling a timeline of all previous depressive episodes using a life history calendar approach.^13^ Parents identified their worst episode, which was rated using the Global Assessment of Functioning (GAF) scale.^14^ The GAF scale ranges from 0 to 100 and provides a measure of social, occupational and psychological functioning, with lower scores indicating greater impairment of functioning. Information about parental depressive episodes occurring during the periods between assessments was collected at waves two and three, allowing lifetime history of parent depression to be collated. History of in-patient admission for depression was also recorded. Semi-structured interviews with the affected parent were completed at each wave of the study. The Schedules for Clinical Assessment in Neuropsychiatry (SCAN)^15^ were used to assess DSM-IV^14^ current depression symptoms in parents and establish whether the parent had experienced an episode of DSM-IV depression over the previous month.

#### Child measures

Child psychopathology was assessed at each wave using the Child and Adolescent Psychiatric Assessment (CAPA),^16^ parent and child versions. This semi-structured research diagnostic interview was used to assess child mood disorders (including major depressive disorder, dysthymia, depressive disorder not otherwise specified (NOS), bipolar affective disorder and cyclothymia), anxiety disorders other than simple phobia (including generalised anxiety disorder, separation anxiety disorder, social anxiety disorder, panic disorder, agoraphobia, obsessive-compulsive disorder and anxiety NOS), disruptive behaviour disorders (including conduct disorder, oppositional defiant disorder, disruptive behaviour NOS) and attention-deficit hyperactivity disorder (ADHD) in the preceding 3 months. At each wave, child research diagnoses were generated according to DSM-IV criteria, based on CAPA symptoms and impairment of functioning. Parent- and child-reported diagnoses were combined and all those meeting criteria, together with subthreshold cases, were reviewed by two child and adolescent psychiatrists. DSM-IV symptom counts were also generated for major depressive disorder, generalised anxiety disorder, ADHD and disruptive behaviour disorder. Symptoms were considered present if reported by either the parent or the child. Psychiatric disorder and symptom counts from the final assessment were used as outcome measures. In addition, a service use interview^17^ was completed by the parent at each wave. This asked about parent or child contact with services (including primary care, paediatrics, mental health services, education, Social Services and youth justice services), in the past 3 months and ever, due to concerns about the child’s emotions or behaviour. Child IQ was assessed at baseline using the Wechsler Intelligence Scale for Children.^18^

#### Family measures

Information about family environment was collected via parent and child questionnaires. Parents completed the Family Environment Scale,^19^ which contains subscales relating to family cohesion and conflict. Children completed the Iowa Family Interaction Rating Scale,^20^ which assesses their perception of warmth and hostility from their mother and father separately.

### Analysis

Rates of child awareness of parent depression were calculated. *t*-tests or χ^2^-tests were used to investigate differences between groups of children rated as aware or unaware on a number of demographic (parent and child gender, parent and child age, income, parental education and child IQ), family (family composition, family cohesion, family conflict, child-rated hostility and lack of warmth from mother, and child-rated hostility and lack of warmth from father) and parental depression characteristics (ever admitted to hospital for depression, worst ever GAF score and current parental depressive episode at final follow-up). Regression analyses were performed to investigate whether child awareness of parental depression was associated with measures of child psychopathology (disorder and symptoms) obtained at the same time point (wave three). Regression analyses were then conducted, adjusting for variables that had been found to be associated with child awareness. All analyses were also run excluding the 18 fathers to establish whether their inclusion affected any associations found. Analyses were performed using SPSS version 18 on Windows XP.

## Results

### Rates of awareness

Nearly all parents in the sample (99.6%) believed someone else to be aware of their depression. They reported a friend was the most likely person to know (83.1%), followed closely by their spouse (82.1%). Seventy-three per cent of parents believed that the study child was aware of their depression, with lower rates for the non-study child (57.6%). Work colleagues were least likely to be aware (40.7%).

**Table 1:** Demographic, family and parent depression characteristics according to child awareness of parental depression

	Child aware (*n*⩽198)^a^	Child unaware (*n*⩽73)^b^	Test statistic (95% CI)	*P*
Demographics				
Child age, years: mean	15.0	14.1	**t** = **3.39 (0.38 to 1.44)**	**0.001**
Child gender, % female	58.4	56.2	χ^2^ = 0.12	0.735
Child IQ, mean	96.5	95.6	*t* = 0.51 (–2.46 to 4.20)	0.609
Parent age, years: mean	44.3	44.0	*t* = 0.40 (–1.15 to 1.80)	0.657
Parent gender, % female	91.8	97.3	χ^2^ = 2.53	0.112
Parent education university or above, %	32.3	36.2	χ^2^ = 0.39	0.532
Parent income <£30 000/per annum, %	53.4	41.4	χ^2^ = 2.70	0.100
One-parent family, %	39.2	39.1	χ^2^ = 0.00	0.989
Parent depression factors				
Worst ever GAF score,^c^ mean	38.0	44.4	**t** = –**2.80 (**–**10.70 to** –**1.89)**	**0.005**
Ever admitted to hospital with depression, %	14.9	6.9	χ^2^ = 2.97	0.085
Depression episode at wave 3, %	18.5	17.8	χ^2^ = 0.40	0.821
*Family factors*				
Child rated, mean				
Hostility from mother	11.5	10.6	*t* = 1.13 (–0.6 to 2.41)	0.259
Lack of warmth from mother	14.3	13.3	*t* = 0.93 (–0.15 to 0.41)	0.350
Hostility from father	11.0	9.4	*t* = 1.70 (–0.03 to 0.45)	0.090
Lack of warmth from father	17.8	15.6	*t* = 1.61 (–0.49 to 4.90)	0.108
Family Environment Scale, mean				
Total	36.4	36.5	*t* = 0.03 (–2.25 to 2.18)	0.975
Conflict	18.2	18.0	*t* = 0.38 (–1.02 to 1.51)	0.703
Cohesion	18.3	18.6	*t* = –0.57 (–1.48 to 0.81)	0.568
Service use ever, %	58.0	45.7	χ^2^ = 3.10	0.078

a. For all variables *n*⩾181, except lack of warmth from father (*n* = 160) and hostility from father (*n* = 161).

b. For all variables *n*⩾69, except lack of warmth from father (*n* = 62) and hostility from father (*n* = 63).

c. Global Assessment of Functioning (GAF) is a measure of social, occupational and psychological functioning in parents during a depressive episode. Lower scores indicate greater impairment in functioning.

Results in bold are statistically significant.

### Differences in the sample according to child awareness of parental depression

The study children who were thought to be aware of their parent’s depression were significantly older than children who were not aware (15.0 *v.* 14.1 years; *t*(267) = 3.39, *P* = 0.001). They were also more likely to be aware when their parent’s depression had been more severe, indexed by a lower GAF score (mean 38.0 *v.* 44.4; *t*(268) = –2.8, *P* = 0.005). There were no other significant differences between the groups in terms of demographic, family or parent depression factors (Table 1). Reported rates of access to services due to concerns about child behaviours or emotions were somewhat higher where children were thought to be aware, although differences failed to reach statistical significance (χ^2^ = 3.10, d.f. = 1, *P* = 0.078). On excluding fathers from the analysis, rates of maternal in-patient admission for depression became significantly greater in the children who were aware (χ^2^ = 4.053, d.f. = 1, *P* = 0.044), but results otherwise remained unchanged (results available on request).

### Associations between child awareness of parental depression and child psychopathology

Associations between child awareness and child psychopathology were assessed. Regression analyses were adjusted for child age and parent worst ever GAF, as these variables were found to be significantly different according to child awareness of parental depression. Rates of disorder were somewhat higher for children reported to be aware of their parent’s depression, although associations between child awareness and child disorder were not significant (Table 2). There was also no significant association between child awareness of parental depression and child symptoms of depression, anxiety, ADHD or disruptive behaviour disorder (Table 2). Excluding fathers from analysis did not affect these findings (results available on request).

## Discussion

### Rates of awareness

Over a quarter of the children participating in this study were thought to be unaware of their parent’s depression, with rates even higher in the siblings not participating. This is despite these families being involved in the EPAD study, specifically studying mood and well-being. It is possible the proportion of children unaware of their parent’s depression would be higher in a sample that had not chosen to take part in such a study.

Although reasons why children may not be aware of their parent’s depression were not explored in this study, feelings of guilt^9^ or concerns about burdening their child with details of their illness^10^ are possible factors. The fact that the parents in this study were less likely to tell their child than others, such as friends or spouses, may support this. This possible reluctance to talk to children in these highly motivated families may reflect how difficult it can be for parents to discuss their depression with their children. It may be that parents would benefit from support from trained professionals in this area.

**Table 2:** Rates of child psychiatric disorder and symptoms according to child awareness of parental depression

			Unadjusted			Adjusted for child age and parent worst ever GAF		
	Child aware^a^	Child unaware^b^	OR (95% CI)	β (95% CI)	*P*	OR (95% CI)	β (95% CI)	*P*
Disorder, % (*n*)								
Mood	11.3 (22)	5.6 (4)	2.16 (0.72 to 6.51)		0.170	1.32 (0.42 to 4.21)		0.637
Anxiety	15.2 (30)	8.2 (6)	2.00 (0.80 to 5.03)		0.141	1.65 (0.63 to 4.30)		0.308
ADHD	1.0 (2)	1.4 (1)	0.73 (0.07 to 8.16)		0.797	0.76 (0.06 to 9.95)		0.831
DBD	9.6 (19)	5.5 (4)	1.84 (0.60 to 5.59)		0.285	1.95 (0.62 to 6.14)		0.253
Any	26.2 (51)	19.2 (14)	1.47 (0.75 to 2.85)		0.259	1.171 (0.58 to 2.40)		0.657
								
Symptoms, mean								
Depressive	2.10	1.64		0.09 (–0.05 to 0.31)	0.148		0.01 (–0.16 to 0.18)	0.905
Anxiety	2.18	1.79		0.06 (–0.12 to 0.30)	0.381		0.01 (–0.19 to 0.24)	0.842
ADHD	0.86	1.19		–0.05 (–0.25 to 0.10)	0.413		–0.05 (–0.26 to 0.11)	0.440
ODD	3.04	2.70		0.07 (–0.08 to 0.28)	0.255		0.06 (–0.10 to 0.27)	0.384

ADHD, attention-deficit hyperactivity disorder; DBD, disruptive behaviour disorder; GAF, Global Assessment of Functioning; ODD, oppositional defiant disorder; OR, odds ratio.

a. For mean symptoms calculation in child aware group *n*⩾185.

b. For mean symptoms calculation in child unaware group *n*⩾68.

### Differences in the sample according to child awareness of parental depression

This study compared characteristics of parents and families according to parent-reported child awareness of parental depression. Children who were thought to be aware of their parent’s depression were significantly older than those children who were not. Parents may worry about telling younger children about their mental illness as they may not understand.^10^ The older the child, the more the parent may feel able to tell them about their difficulties. Despite this, all children included in this study were aged 9 years or older, and resources do exist (leaflets, websites, books or films, e.g. Children of Parents with a Mental Illness, www.copmi.net.au; Royal College of Psychiatrists^21^) for children of this age group to help them understand parental mental illness.

Impairment in functioning during the parent’s worst depressive episode was also associated with parent-reported child awareness. Levels of impairment could be seen as a marker of depression severity and it may be more difficult for a parent to keep their depression from their child if it is severe. For example, it may be more apparent that an individual has depression if they are unable to function, require admission to hospital or input from a community mental health team. Being aware of their parent’s depression in these circumstances does not necessarily mean the child has a full understanding of their parent’s illness and just suspecting that something is wrong, without understanding, may be more difficult for the child than having no awareness at all. Higher rates of awareness in this group may be an indication that families with parents with severe depression may benefit from support to help their child to understand their illness.

No other demographic, family or parental depression factors investigated were found to be significantly associated with child awareness. One might have expected that family environment factors such as cohesion or increased warmth would have an impact on how open families are with one another about sensitive issues, but we did not find evidence in support of this. The child-rated hostility and lack of warmth from the father was higher in children who were aware of their parent’s depression, but levels did not reach significance. However, these results must be considered in the light of the fact that we did not obtain information on how, why or when the child became aware of their parent’s depression or their level of awareness.

### Associations between child awareness of parental depression and child psychopathology

Associations between child awareness of parental depression and child psychopathology were also examined. We did not find any significant differences in rates of psychiatric disorder or symptoms between children who knew about their parent’s depression compared with those who did not, even when adjusted for child age and depression severity.

Although results in this study did not show any significant association between child awareness of parental depression and psychopathology, it is worth noting that the percentage of children with mood disorder, anxiety disorder and disruptive behaviour disorder were higher in those who knew about their parent’s depression than those who did not. It is possible that associations might arise through selection rather than risk effects; for example where parental disclosure to children is influenced by the presence or absence of child psychopathology. If a child develops depression, the parent may be more likely to tell them they have had depression too. The results also showed a trend of increased rate of access to services in children who were thought to be aware of their parent’s depression. It is possible that if the child has required input from services, the parents may be more inclined to tell them about their own difficulties. Even so, our findings do not support evidence in favour of disclosure being associated with either risks or benefits in terms of child psychopathology.

Other studies have explored parental disclosure to children in the context of other disorders. They highlight the benefits of talking openly about illness within families. Children want to understand and have knowledge of their parent’s illness and may react negatively to lack of information.^22,23^ However, others exploring topics unrelated to illness, have suggested that discussing sensitive topics may lead to adolescents worrying about their parents’ well-being.^23^ Koerner *et al*^24^ looked at disclosure by mothers to adolescent daughters regarding financial concerns and complaints of anger towards their ex-husband. They suggested that children may feel overwhelmed by such knowledge. Lichtwarck-Aschoff *et al*^25^ found that disclosure of similar stressful information to children resulted in more externalising and internalising problems. Therefore, what is being disclosed to a child may have an impact on how it affects them.

### Limitations

The study provides information in an area where there is limited existing literature, but the results must be interpreted in light of several limitations. First, as mentioned earlier, we do not know how, when or why the child was made aware of their parent’s depression, or how much was disclosed. If they became aware out of necessity rather than choice, this may have an impact on the effect this knowledge has on them. For example, becoming aware because a parent is admitted to hospital may have a different impact on the child compared with a disclosure following a planned discussion. Second, child awareness of parental depression was rated by the affected parent rather than the child. It is possible that some children may have been aware without their parents’ knowledge, whereas others may have known less than their parents suspected. Another limitation is that this study sample may not be representative of a general population of parents with depression. These parents chose to take part in a study of mood and well-being in families involving their children, and so may be more open about sharing their diagnosis. Finally, with relatively small numbers of children developing each disorder, it is possible that a larger sample would provide more power to detect associations between child awareness of parental depression on child psychopathology.

### Clinical implications

Clinicians working with parents with depression should consider the wider family - and issues including disclosure - because of the possible impact on the child. Although it is ultimately the parents who will decide whether or not to tell their children about their mental illness, they may benefit from discussions and support from trained professionals in order to consider the consequences of doing so.

In addition, children of parents with depression are at increased risk of experiencing depression themselves^26^ and targeted early intervention and prevention strategies are a priority for them. If a child is not aware of their parent’s depression, this may be a barrier to taking up these initiatives. It may be easier to identify and engage children in prevention programmes and family interventions if they are aware of their parent’s illness.
